# I-*Sce*I-Mediated Double-Strand Break Does Not Increase the Frequency of Homologous Recombination at the *Dct* Locus in Mouse Embryonic Stem Cells

**DOI:** 10.1371/journal.pone.0039895

**Published:** 2012-06-26

**Authors:** Myriam Fenina, Dominique Simon-Chazottes, Sandrine Vandormael-Pournin, Jihane Soueid, Francina Langa, Michel Cohen-Tannoudji, Bruno A. Bernard, Jean-Jacques Panthier

**Affiliations:** 1 Mouse functional Genetics, Institut Pasteur, Paris, France; 2 CNRS URA 2578, Institut Pasteur, Paris, France; 3 Mouse Genetics Engineering Center, Institut Pasteur, Paris, France; 4 Life Sciences Department, L’Oréal Recherche and Innovation, Clichy, France; Ohio State University Comprehensive Cancer Cente, United States of America

## Abstract

Targeted induction of double-strand breaks (DSBs) at natural endogenous loci was shown to increase the rate of gene replacement by homologous recombination in mouse embryonic stem cells. The gene encoding dopachrome tautomerase (*Dct*) is specifically expressed in melanocytes and their precursors. To construct a genetic tool allowing the replacement of *Dct* gene by any gene of interest, we generated an embryonic stem cell line carrying the recognition site for the yeast I-*Sce*I meganuclease embedded in the *Dct* genomic segment. The embryonic stem cell line was electroporated with an I-*Sce*I expression plasmid, and a template for the DSB-repair process that carried sequence homologies to the *Dct* target. The I-*Sce*I meganuclease was indeed able to introduce a DSB at the *Dct* locus in live embryonic stem cells. However, the level of gene targeting was not improved by the DSB induction, indicating a limited capacity of I-*Sce*I to mediate homologous recombination at the *Dct* locus. These data suggest that homologous recombination by meganuclease-induced DSB may be locus dependent in mammalian cells.

## Introduction

The natural efficiency of the introduction of defined sequences at specific locations of the mouse genome in embryonic stem (ES) cells by homologous recombination (HR) varies between 10^−5^ and 10^−8^ events per treated cell. Such a frequency is too low to consider the iterative introduction of a number of genes of interest at a given locus in standard practice. This problem can be overcome by enhancing recombination reactions at the target site through the induction of a double-strand break (DSB) [1]. Such DSBs can be induced with the yeast mitochondrial I-*Sce*I meganuclease which has an 18-bp recognition site, absent normally in the mammalian genome but that may be added to the genome of recipient cells. In previous studies performed with I-*Sce*I in Chinese hamster ovary (CHO) cells, mouse 3T3 fibroblasts, PCC7-S multipotent cells, and also in several ES cell lines, specific DSBs were shown to stimulate the repair of a tandem duplication by intrachromosomal HR or gene targeting by plasmid-to-chromosome HR [2]–[8]. More recently, plasmid-mediated gene targeting was achieved in CHO cells after lentiviral delivery of the I-*Sce*I protein [9]. Expression of I-*Sce*I was also shown to be relevant to improve the efficiency of gene targeting in other organisms, including flies and plants [10], [11]. Based on these data, it is generally admitted that a system based on the introduction of an I-*Sce*I recognition site close to the locus to be targeted in the genome of recipient ES cells, combined with transient expression of the I-*Sce*I meganuclease to create a DSB, should enhance the introduction of donor sequences at this site.

The *Dct* gene encodes the dopachrome tautomerase, a melanogenic enzyme. In the embryo, *Dct* is expressed in pigment cell precursors, i.e. melanoblasts, derived from the neural crest, in cells of the retinal pigment epithelium and in the developing forebrain [12], [13]. In the adult epidermis, *Dct* is expressed in pigment cells at all differentiation states: in stem cells that reside in the bulge region of the hair follicle, in progenitors of the outer root sheath and in melanocytes of the hair matrix [14]. Studies performed in the mouse embryo with a *LacZ* reporter gene expressed under the control of 3.4 kb of the *Dct* promoter (*Dct-LacZ* transgene) depicted *LacZ* expression in melanoblasts and melanocytes, in the retinal pigment epithelium, forebrain, dorsal root ganglia and caudal nerves [15]. In addition, the *Dct-LacZ* reporter allowed to monitor cells of the melanocyte lineage in adult mice [16]. Altogether, *Dct* promoter-driven expression was shown to mimic largely the endogenous expression pattern of the gene. The *Dct* promoter has thus been used to drive the expression of genes in melanocytes and their precursors in transgenic mice [17]–[21]. However the use of combination of regulatory region from *Dct* and the coding regions of exogenous genes, either reporter genes or genes whose function is to be evaluated, can also have large drawbacks. First, several independent lines are required to distinguish the specific expression of the transgene from ectopic expression. Second, the transgene may be expressed in tissues that do not normally express endogenous *Dct*. This has been repeatedly observed with the *Dct* promoter [15], [17], [22]. It is worth noting that *Dct* knockout mice are viable and fertile, and exhibit no defects, with the exception of a diluted coat colour [23], making *Dct* an interesting driver to monitor the effects of expression of genes of interest, such as genes that may be involved in melanoma progression [24], [25]. Indeed, either homozygous knockin mice or double heterozygotes for a reporter gene and the gene of interest may be studied. We thus became interested by constructing a genetic tool that would allow to insert with a high efficiency any gene of interest in place of the *Dct* gene.

Our approach relied on the combination of three components: an ES cell line carrying a *Dct* allele with the I-*Sce*I recognition site, an I-*Sce*I expressing plasmid and a template for the DSB-repair process carrying sequence homologies to the *Dct* locus. The targeted integration at the *Dct* locus was tested after transfection of both the I-*Sce*I expressing plasmid and the repair construct in the modified ES cell line. We report here that an I-*Sce*I recognition site embedded within the *Dct* gene sequence can be cleaved by transiently expressed I-*Sce*I meganuclease in ES cells. We further show, contrary to expectations, and using two different repair vectors, that I-*Sce*I-mediated DSB did not increase the frequency of HR at the *Dct* locus compared to conventional gene targeting experiments.

## Materials and Methods

### Ethics Statement

Animals were housed in animal facilities accredited by the French Ministry of Agriculture to perform experiments on live mice, in appliance of the French and European regulations on care and protection of the Laboratory Animals (accreditation number B 75 15-01 and B 75 15-07). The veterinary staff of the Institut Pasteur animal facility approved protocols. Protocols were performed in compliance with the NIH Animal Welfare Insurance #A5476-01 issued on 02/07/2007.

### Plasmids and Cells

Plasmid pPBSKλB#4 was given by I.J. Jackson (MRC, Edinburgh, UK). Plasmids pL253, pL452, pSW23 were provided by N. Copeland and N. Jenkins (Frederick, MD, USA). To obtain pCAG-I-*Sce*I, the enhancer of the major immediate-early enhancer of the human cytomegalovirus (CMV) contained in pCMV-I-*Sce*I [26] was replaced by the chicken β-actin promoter and cytomegalovirus enhancer [27]. CK35 ES cells [28] were grown on mitomycin C-treated Neo^R^ primary fibroblasts in Dulbecco’s modified Eagle’s Medium + GlutaMAX (Invitrogen) supplemented with 15% fetal bovine serum, 0.1 mM β-mercaptoethanol (Sigma), 10^3^ U/ml murine LIF (PAA Laboratories) as previously described [29].

### Design of Repair Vectors

To insert an I-*Sce*I recognition site at the *Dct* locus, a replacement vector was constructed. A 6.5 kb *Snab*I-*Hinc*II fragment from pPBSKλB#4 that contains 18 kb of *Dct* gene (MGI:102563) [30] was inserted into the *Spe*I site of pL253 to produce pL253-*Dct* plasmid. To introduce an I-*Sce*I recognition site and a Neo^R^ cassette into the 6.5 kb *Dct* fragment near the first exon, we took advantage of a unique *Nhe*I site located within intron 1, 112 bp downstream of the first exon. A 5′ *Dct* fragment containing the *Nhe*I site was amplified and flanked with *Kpn*I and *Eco*RI sites using the following primers: 5′*Kpn*I forward 5′-ATAGGTACCTCCCAATTAAGAAGGCATGG-3′ and 5′*Eco*RI reverse 5′-GCGGAATTCCGCCTTTCTGAGTGAAGAG-3′. The amplicon was inserted into pCR2.1 plasmid (TOPO TA Cloning®, Invitrogen). The I-*Sce*I recognition site was added at the *Nhe*I site. Besides, a 3′ *Dct* fragment was amplified and flanked with *Bam*HI and *Sac*II sites using the following primers: 3′*Bam*HI forward 5′-GTAGGATCCACCTTTGGCTTGTTTGTTGG-3′ and 3′*Sac*II reverse 5′-ATACCGCGGAGGACATGAGAACCCCAGA-3′. The amplicon was inserted into a pCR2.1 plasmid. pSW23 plasmid was digested by *Kpn*I and *Sac*II, and filled in with the three fragments: the 5′ *Kpn*I-*EcoR*I fragment containing the I-*Sce*I site, an *Eco*RI-*Bam*HI Neo^R^ cassette from pL452 plasmid and the 3′ *Bam*HI-*Sac*II fragment. The replacement vector was produced by a recombineering reaction between the modified pSW23 and pL253-*Dct* plasmid. The replacement vector carries a herpes simplex virus-thymidine kinase (HSV-TK) negative selection cassette downstream of the 6.5 kb *Dct* fragment.

**Figure 1:**
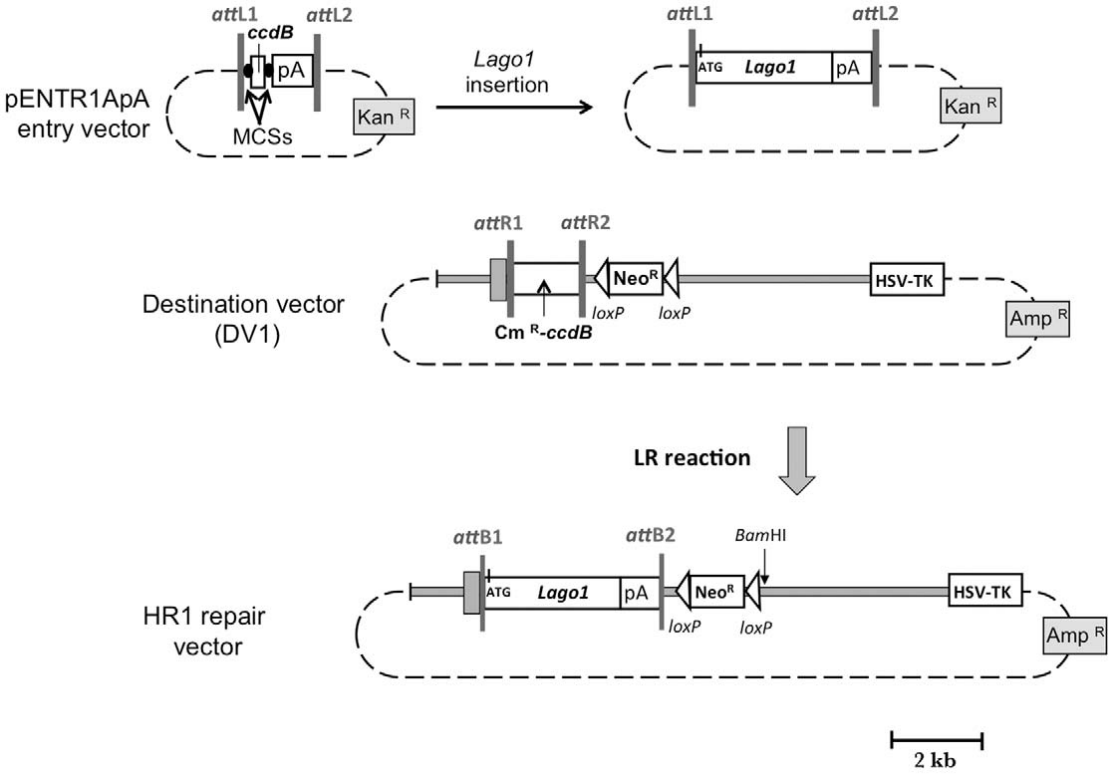
Construction of the HR1 repair vector. The pENTR1ApA entry vector is represented in the upper left. Black circles flanking *ccdB* represent multi-cloning sites (MCSs). The SV40 polyadenylation sequence (pA) was introduced at the 3′ end of the *ccdB* gene, before *att*L2 sequence. The entry vector contains a Kan^R^ cassette conferring resistance to kanamycin in *E. coli*. The entry vector carrying *Lago1* gene is represented in the upper right. The destination vector (DV1) contains 1.4 and 4.5 kb of *Dct* homologous arms depicted as grey rectangles. DV1 also contains the Neo^R^ and HSV-TK cassettes used in cell culture, and an Amp^R^ cassette conferring resistance to ampicillin in *E. coli*. The repair vector (HR1) is produced by LR reaction, allowing the replacement of Cm^R^-*ccdB* cassette by the *Lago1* gene.

The construction of HR repair vectors HR1 and HR2 relied on the Gateway® technology (Invitrogen). Entry and destination vectors were produced. pENTR1A entry vector (Invitrogen) contains *ccdB* flanked with multi-cloning sites (MCS). The SV40 polyadenylation sequence (pA) was inserted at the *Eco*RV site to give pENTR1ApA. The *Lago1* gene, a synthetic CpG-free *LacZnls* reporter gene that contains a SV40 nuclear localization signal (Invivogen), with its start codon was inserted in pENTR1ApA in place of *ccdB* (Fig. 1). The first destination vector (DV1) was constructed as follows. Starting from pL253-*Dct* plasmid, a *Sex*AI-*Avr*II 400 bp fragment containing the ATG start codon was removed from *Dct* sequence. A linker made of the following primers was used to fill the gap in *Dct* sequences: HpaPme forward 5′-CTAGGTTAACGTTTAAA-3′ and HpaPme reverse 5′-CCTGGTTTAAACGTTAAC-3′. The linker allowed the insertion of a unique *Hpa*I recognition site into pL253-*Dct*, giving pL253-*Dct*-*Hpa*I plasmid. To insert the Neo^R^ cassette into pL253-*Dct*-*Hpa*I, a recombineering reaction was performed and gave pL253-*Dct*-*Hpa*I-Neo^R^ plasmid. Finally, a reading frame cassette A (RfA) (Gateway® technology) that contains the Cm^R^-*ccdB* cassette flanked by *att*R1 and *att*R2 sites, was inserted at the *Hpa*I site into pL253-*Dct*-*Hpa*I-Neo^R^ to obtain DV1. In DV1, the negative selection cassette HSV-TK from pL253 is downstream of the 3′ sequence homology of *Dct* (Fig. 1). To produce the first repair vector (HR1), the Cm^R^-*ccdB* cassette in DV1 was replaced by *Lago1* gene using LR reaction (Gateway® technology) (Fig. 1).

**Figure 2:**
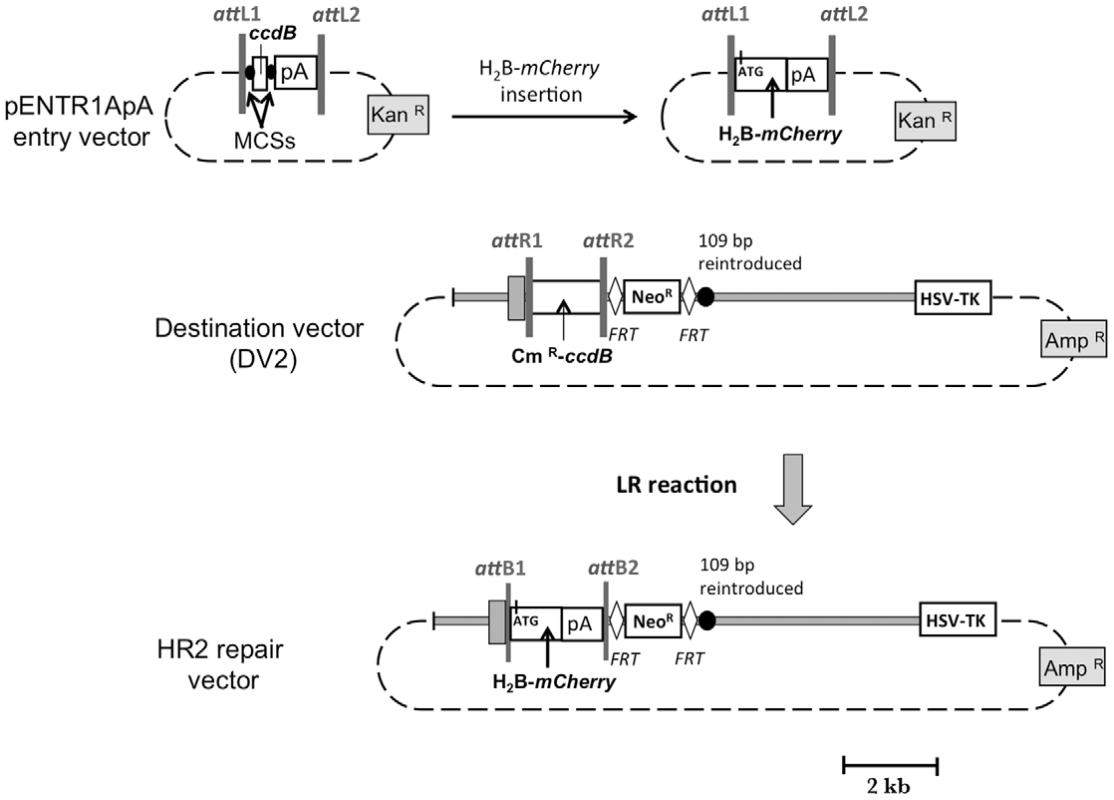
Construction of the HR2 repair vector. The pENTR1ApA entry vector is represented in the upper left. The entry vector containing the H_2_B-*mCherry* gene is represented in the upper right. The DV2 destination vector contains the *Dct* homologous arms, 1.4 and 4.5 kb in length, depicted as grey rectangles. The black circle denotes 109 bp of *Dct* intron absent in DV1 destination vector that were inserted in DV2 destination vector. DV2 also contains a Neo^R^ cassette flanked with *FRT* sites depicted as white diamond symbols. The repair vector (HR2) is produced by LR reaction, allowing the replacement of Cm^R^-*ccdB* cassette by the H_2_B-*mCherry* gene.

To remove the short regions of homology between the HR1 repair vector and the first intron of *Dct* gene, a second destination vector (DV2) was produced. The H_2_B-*mCherry* reporter gene with its start codon [31] was inserted in place of *ccdB* in pENTR1ApA entry vector (Fig. 2). To obtain DV2, a 2.8 kb *Avr*II-*Bsu*36I fragment was synthetized. It contains a Neo^R^ cassette framed with *FRT* sites and 1 kb of *Dct* genomic sequence. A 1 kb *Avr*II-*Bsu*36I fragment was removed from the pL253-*Dct*-*Hpa*I plasmid and replaced by the 2.8 kb synthetized *Avr*II-*Bsu*36I fragment. Then RfA was ligated at the *Hpa*I site. Figure 2 shows the map of DV2. To produce the second repair vector (HR2), the Cm^R^-*ccdB* cassette of DV2 was replaced by the H_2_B-*mCherry* sequences using LR reaction (Fig. 2). The integrity of the repair vectors was verified by sequence analysis.

### Homologous Recombination Assay

To insert a unique I-*Sce*I site at the *Dct* locus, approximately 1.6 × 10^7^ CK35 ES cells were electroporated with the *Not*I-linearized replacement vector. G418 (300 µg/mL) was added 48 h after plating for 12 days and gancyclovir (2 µM) was added 96 h after plating for 4 days. The Neo^R^ cassette was removed using pIC-Cre plasmid [32] in which the transcription of Cre recombinase is driven by a synthetic HSV-TK promoter and enhancer. Fifteen micrograms of pIC-Cre plasmid were electroporated into approximately 1×10^7^ ES clone 4 cells and the cells were cultured without G418. For the gene targeting with HR1 repair vector, approximately 1.3×10^7^ MF1 ES cells were electroporated with 20 µg of supercoiled HR1 alone or with 33 µg pCMV-I-*Sce*I [26] or 37.5 µg pCAG-I-*Sce*I. The ratio of expression plasmid to repair vector was 5 to 1. For the experiment using HR2, approximately 1.6×10^7^ MF1 ES cells were electroporated with 30 µg of supercoiled HR2 plasmid alone or with either 11 µg pCMV-I-*Sce*I or 13 µg pCAG-I-*Sce*I expression plasmids. The ratio of expression plasmid to repair vector was 1 to 1. Approximately 1.6×10^7^ MF1 ES cells were independently electroporated with 30 µg of *Not*I-linearized HR2 plasmid, as a control.

### DNA Analysis in Selected Clones

Genomic DNAs of ES clones obtained after selection with G418 and gancyclovir were digested with *Bam*HI. Correct gene targeting was analyzed by Southern blot using a 1 kb 5′ external probe produced by PCR amplification with the following primers: 5DCTF forward 5′-TTGGGGTCAGGGAGATACAG-3′ and 5DCTR reverse 5′-TGAGCAGCAGTGAAGTTTGG-3′.

#### Generation of *Dct^I-SceI^* ^/+^ Mice

Two independent *Dct^I-SceI^* ^/+^129/Sv ES clones, named MF1 and MF2, were introduced into C57BL/6N blastocysts, which were transferred to pseudo-pregnant C57BL/6N females. Male chimeras (129/Sv *Dct^I-SceI^* ^/+^ <-> C57BL/6N *Dct ^+/+^*) were mated with C57BL/6N females. The progeny was genotyped at the *Dct* locus to evaluate the percentage of *Dct^I-SceI^* ^/+^ mice.

### Ligation-Mediated PCR (LM-PCR) Analysis

Approximately 1.6 × 10^7^ MF1 ES cells were electroporated with 50 µg pCMV-I-*Sce*I, pCAG-I-*Sce*I or mock plasmid. Four hours later, genomic DNA was extracted. Two micrograms of genomic DNA from MF1 cells transfected with the mock plasmid were digested with *Pst*I or I-*Sce*I and precipitated. LM-PCRs were performed with these *Pst*I- and I-*Sce*I-digested DNAs, and with undigested DNA from MF1 cells transfected with mock plasmid, pCMV-I-*Sce*I or pCAG-I-*Sce*I. The specific LM-C1 primer 5′-AATTCTTCAACCGGACAT-3′ was used for the first extension. The asymmetrical synthetic double-stranded linker was prepared by hydridization of two oligonucleotides: linkerF forward 5′-GCGGTGACCCGGGAGATCTGAATTC-3′ and reverse linkerR 5′-GAATTCAGATC-3′. The specific LM-C2 primer 5′-CGGACATGCAAATGCACAGGTGAGG-3′ was used for a first PCR amplification. The PCR product was subjected to nested PCR with the specific LM-C3 primer 5′-CCCTTGGGCAGACCCAGATGTCACT-3′) and linkerF. After agarose gel electrophoresis and alkaline transfer to a nylon membrane, the DNA was hybridized to the specific 36-mer radioactive probe LM 5′-CTTCTGAGGAGAGGCGACACTGGTGACAAACTGTTA-3′.

## Results

Our experiments aimed at testing the efficiency of a ready-to-use tool to produce ES cells, and eventually mice, carrying any sequence of interest inserted in place of the *Dct* gene. Our strategy relied on the reported stimulation of gene targeting frequency at a natural locus associated with a DSB induced by the yeast meganuclease I-*Sce*I in ES cells [3]. We performed a two-step experiment. In a first step, the I-*Sce*I restriction site was inserted to the *Dct* gene in ES cells using conventional gene targeting procedures. The *Dct* gene carrying a unique I-*Sce*I restriction site was thus considered as a preferential target for HR. In a second step, an I-*Sce*I-expression plasmid was introduced together with a repair vector sharing a 5.9-kb of *Dct* isogenic DNA by electroporation in the engineered ES cells, and the efficiency of gene targeting at the *Dct* locus was assayed.

### Production of a New Target Allele at the *Dct* Locus in ES Cells

**Figure 3:**
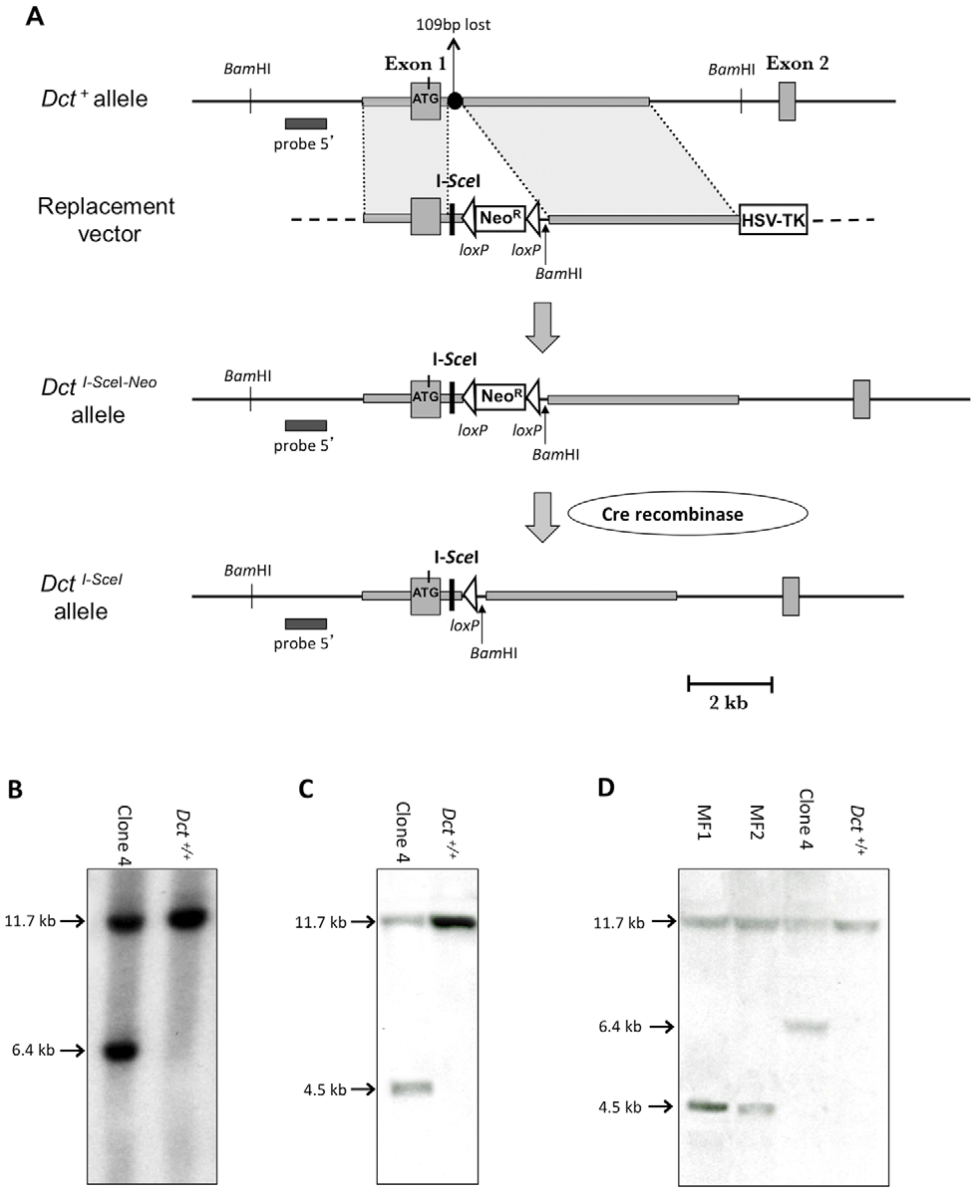
Production of a new target allele at the *Dct* locus. (A) Introduction of an I-*Sce*I site at the *Dct* locus. From top to bottom are represented the *Dct* wild-type allele (*Dct^+^*), the replacement vector, the *Dct^I-SceI-Neo^* targeted allele, and the *Dct^I-SceI^* allele produced after deletion of the Neo^R^ cassette. The grey boxes represent exons 1 and 2 of the *Dct* gene. The black circle represents 109 bp of *Dct* intron 1 sequence that are lost during an homologous recombination event. The horizontal black bar represents the external 5′ probe used for the Southern blots. The Neo^R^ and HSV-TK cassettes are depicted as white rectangles. *loxP* sites are represented by white triangles. The *Dct* homologous arms, 1.9 and 4.5 kb in length, are denoted as grey rectangles. I-*Sce*I and *BamH*I restriction sites are indicated. (B) Southern blot analysis of *Dct^+/+^* ES cells and targeted ES cells (clone 4). Genomic DNAs of ES cells were digested with *Bam*HI. The 11.7 and 6.4 kb fragments are distinctive of the *Dct^+^* and *Dct^I-SceI-Neo^* alleles, respectively. (C) Test of the ability of I-*Sce*I meganuclease to specifically cleave *Dct^I-SceI-Neo^* ^/+^ ES cells. Southern blot analysis of *Dct^+/+^* ES cells and clone 4. Genomic DNAs were digested with I-*Sce*I and *Bam*HI. The 4.5 kb fragment is distinctive of the *Dct^I-SceI-Neo^* allele. (D) Southern blot analysis of *Dct^+/+^* ES cells, clone 4, MF1 and MF2 clones. Genomic DNAs were digested with *Bam*HI. The 4.5 kb fragment is distinctive of the *Dct^I-SceI^* allele.

As a first step, the I-*Sce*I restriction site was inserted within *Dct* intron 1 in ES cells. A replacement vector containing a unique I-*Sce*I restriction site, a positive selection (Neo^R^) cassette flanked by *loxP* sites, and 1.9 and 4.5 kb of 5′ and 3′ genomic sequences from the *Dct* gene was constructed. A negative selection cassette (HSV-TK) was added after the 3′ homology arm (Fig. 3A). The replacement vector was linearized and electroporated into CK35 ES cells. The cells were cultured in the presence of G418 and gancyclovir. Out of 107 G418- and gancyclovir-resistant colonies, one clone (ES clone 4) was correctly targeted with the replacement vector as shown by PCR (data not shown), and later confirmed by Southern blot analysis (Fig. 3B). To test whether the meganuclease I-*Sce*I is able to specifically cleave the new *Dct^I-SceI-Neo^* allele, genomic DNA of the ES clone 4 was treated with both *Bam*HI and I-*Sce*I restriction enzymes. Southern blot analysis using an external 5′ probe revealed the 4.5 kb *Bam*HI-I-*Sce*I distinctive fragment, indicating that the I-*Sce*I site inserted at the *Dct* locus was indeed cut *in vitro* by the meganuclease (Fig. 3C).

To delete the Neo^R^ cassette, a Cre recombinase-expressing plasmid (pIC-Cre) was electroporated into clone 4 *Dct^I-SceI-Neo^* ^/+^ ES cells. A preliminary experiment indicated that more than 30% of the cells transfected with pIC-Cre plasmid died in presence of G418, presumably because they had lost the Neo^R^ cassette. pIC-Cre plasmid was electroporated into *Dct^I-SceI-Neo^* ^/+^ ES cells and the cells were cultured without G418. Twenty-four clones were picked up and their sensitivity was assessed by adding G418 on a duplicate plate: 8 clones were Neo^S^. All eight clones had lost the Neo^R^ cassette as shown by PCR analysis (data not shown). Two ES clones (MF1 and MF2) were further selected on morphological criteria. Southern blot analysis further confirmed the deletion of the Neo^R^ cassette in MF1 and MF2 clones (Fig. 3D).

To test whether *Dct^I-SceI^* ^/+^ MF1 and MF2 clones are able to colonize the germ line, we injected MF1 and MF2 cells into C57BL/6N blastocysts, and thereafter transferred the embryos to pseudo-pregnant females. Twelve and ten chimeras were produced from MF1 and MF2 cells, respectively. Altogether 18 chimeras were more than 95% chimeric, based on their coat colour pattern. Several male chimeras were mated to C57BL/6N females. Half of their progeny was *Dct^I-SceI^* ^/+^, indicating that the genome of MF1 and MF2 ES cells was transmitted via the germ line.

### Insertion of *Lago1* at the *Dct* Locus

**Figure 4:**
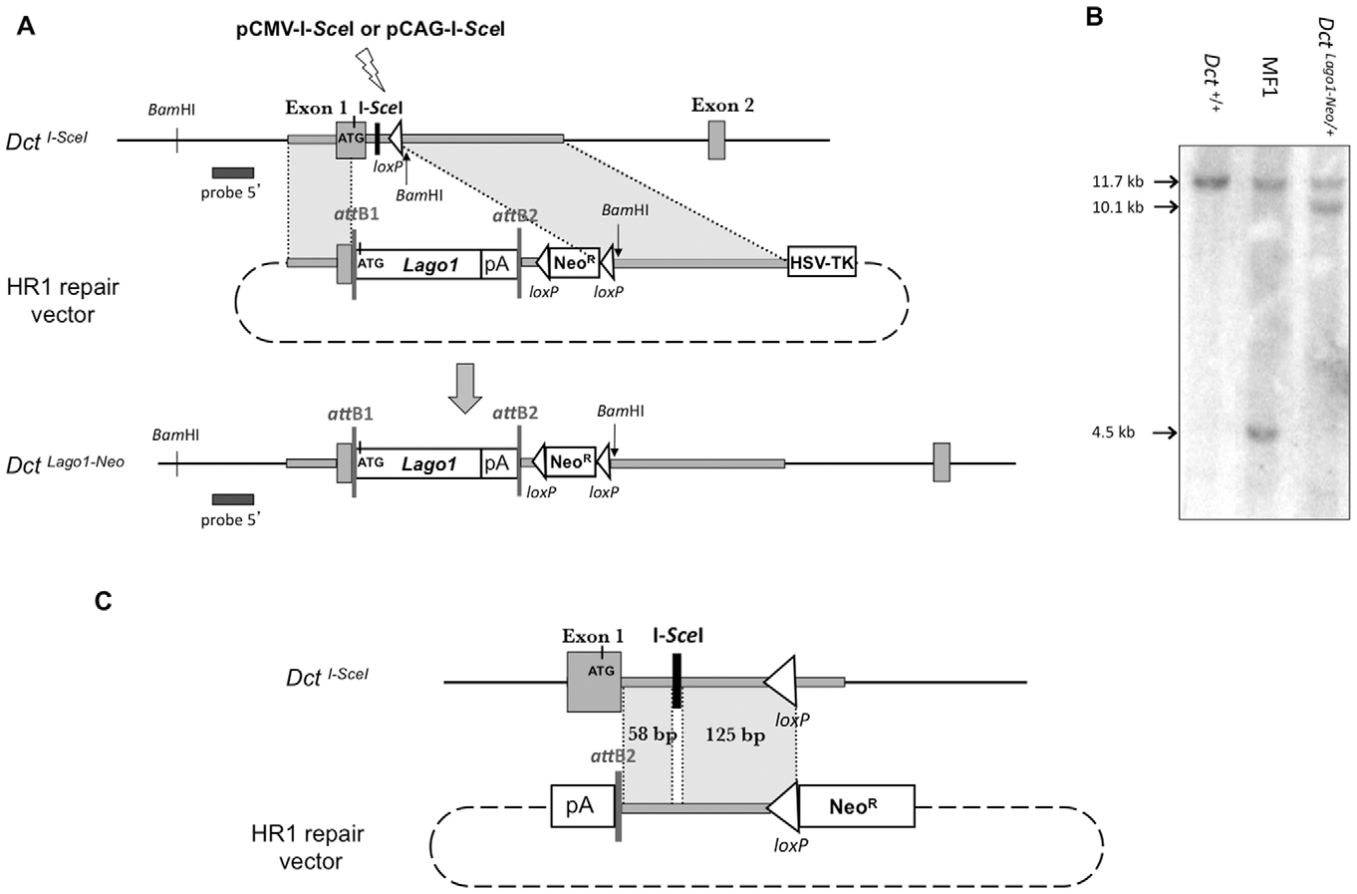
*Lago1* gene targeting by HR at the *Dct* locus. (A) Insertion of *Lago1* gene at the *Dct* locus. The *Dct^I-SceI^* allele, the HR1 repair vector and the *Dct^Lago1^* ^-*Neo*^ targeted allele are represented from top to bottom. A lightning denotes I-*Sce*I expression from pCMV-I-*Sce*I or pCAG-I-*Sce*I plasmid. The 1.4 and 4.5 kb of *Dct* isogenic DNA are depicted by grey rectangles. (B) Southern blot analysis of *Dct^+/+^*, *Dct^I-SceI/+^* and *Dct^Lago1^* ^-*Neo/+*^ ES cells. Genomic DNAs were digested with *Bam*HI. The probe used for the hybridization is the external 5′ probe depicted by a black bar. The 11.7, 4.5, and 10.1 kb fragments are distinctive of the *Dct^+^*, *Dct^I-SceI^*, and *Dct^Lago1^* ^-*Neo*^ alleles, respectively. (C) Diagram of DSB-induced homologous recombination with no insertion of the *Lago1* gene. The *Dct^I-SceI^* allele, and the HR1 repair vector are represented from top to bottom. The short regions of homology, 58 bp and 125 bp in length, between the HR1 repair vector and the genomic DNA at the *Dct* locus in *Dct^I-SceI/+^* cells, are depicted by grey rectangles. An HR between these two short regions of homology would lead to loss of the I-*Sce*I site with no integration of the Neo^R^ cassette. The resulting cells would die in the presence of G418.

We wished to repeatedly introduce gene of interest at the *Dct* locus. As a first attempt, we used the *Lago1* gene. The HR1 repair vector contained *Lago1*, a Neo^R^ cassette framed with *loxP* sites, two regions of homology with the *Dct^I-SceI^* allele, 1.4 and 4.5 kb in length, and a HSV-TK negative selection cassette (Fig. 4A). The construction of the HR1 repair vector relied on the Gateway® technology (see Materials and Methods, and Fig. 1). We assessed the rate of insertion of *Lago1* gene at the *Dct* locus following DSB-induced HR. MF1 ES cells were electroporated with supercoiled HR1 either with or without an I-*Sce*I expressing plasmid. Two different I-*Sce*I-expressing plasmids were tested: (i) pCMV-I-*Sce*I in which I-*Sce*I expression is driven by the cytomegalovirus promoter [3], [26], and (ii) pCAG-I-*Sce*I, where I-*Sce*I is expressed under the control of the CAG composite promoter (see Materials and Methods). The cells were exposed to G418 and gancyclovir. A total of 215, 235 and 252 colonies resistant to both antibiotics were obtained when MF1 ES cells were transfected with HR1 alone, and in combination with pCMV-I-*Sce*I or pCAG-I-*Sce*I respectively (Table 1). For each experiment, 136 colonies were individually picked up and PCR tested. Transfection with either HR1 alone or with HR1 and pCMV-I-*Sce*I gave no targeted colonies. Transfection with HR1 and pCAG-I-*Sce*I gave a positive PCR signal (data not shown), which was confirmed by Southern blot analysis (Fig. 4B and data not shown). Thus gene targeting using the HR1 repair vector and pCAG-I-*Sce*I led to a frequency of HR that could be estimated at 1.4 × 10^−7^ events per treated cell. This frequency is not higher than that obtained with the conventional gene targeting procedures (generally range between 10^−5^ and 10^−8^ events per treated cell).

**Table 1:** Frequency of homologous recombination at the *Dct* locus.

Repair vector and conformation	I-*Sce*I-expressing plasmid	Total electroporated cells	Total G418^r^ gancyclovir^r^	Analyzed G418^r^ gancyclovir^r^	Targeted integration	Gene targeting frequency
**HR1 circular**	None	13×10^6^	215	136	0	0
	CMV-I-*Sce*I	13×10^6^	235	136	0	0
	CAG-I-*Sce*I	13×10^6^	252	136	1	1.4×10^−7^
**HR2 circular**	None	16×10^6^	653	144	0	0
	CMV-I-*Sce*I	16×10^6^	397	144	0	0
	CAG-I-*Sce*I	16×10^6^	370	144	1	1.6×10^−7^
**HR2 linear**	None	16×10^6^	635	144	1	2.7×10^−7^

Elliott and colleagues (1998) reported previously that, in I-*Sce*I-induced gene targeting with a transfected circular plasmid, the majority of recombination events occurred within 100 bp from the cleavage site [33]. Actually, the HR1 repair vector contains two short regions of homology with the targeted *Dct^I^* ^-*SceI*^ allele next to the I-*Sce*I site. These regions are shown in Figure 4C. In the *Dct^I-SceI^* allele, the first short region of homology is located between exon 1 of the *Dct* gene and the I-*Sce*I site. It encompasses 58 bp of *Dct* intron 1 sequence. Still in the *Dct^I-SceI^* allele, a second region of homology is located between I-*Sce*I site and the end of *loxP* site. It encompasses 125 bp of *Dct* intron 1 sequence and *loxP* sequence. In HR1 repair vector, both 58 bp and 125 bp regions are located between the *attB2* site and the Neo^R^ cassette. We hypothesized that these homology regions, 183 bp in total length, could be used as an efficient repair template and would produce by HR a recombinant allele harbouring neither a *Lago1* gene nor a Neo^R^ cassette (Fig. 4C). Hence, clones that have undergone HR would die in the presence of G418.

### Insertion of *H_2_B-mcherry* at the *Dct* Locus

**Figure 5:**
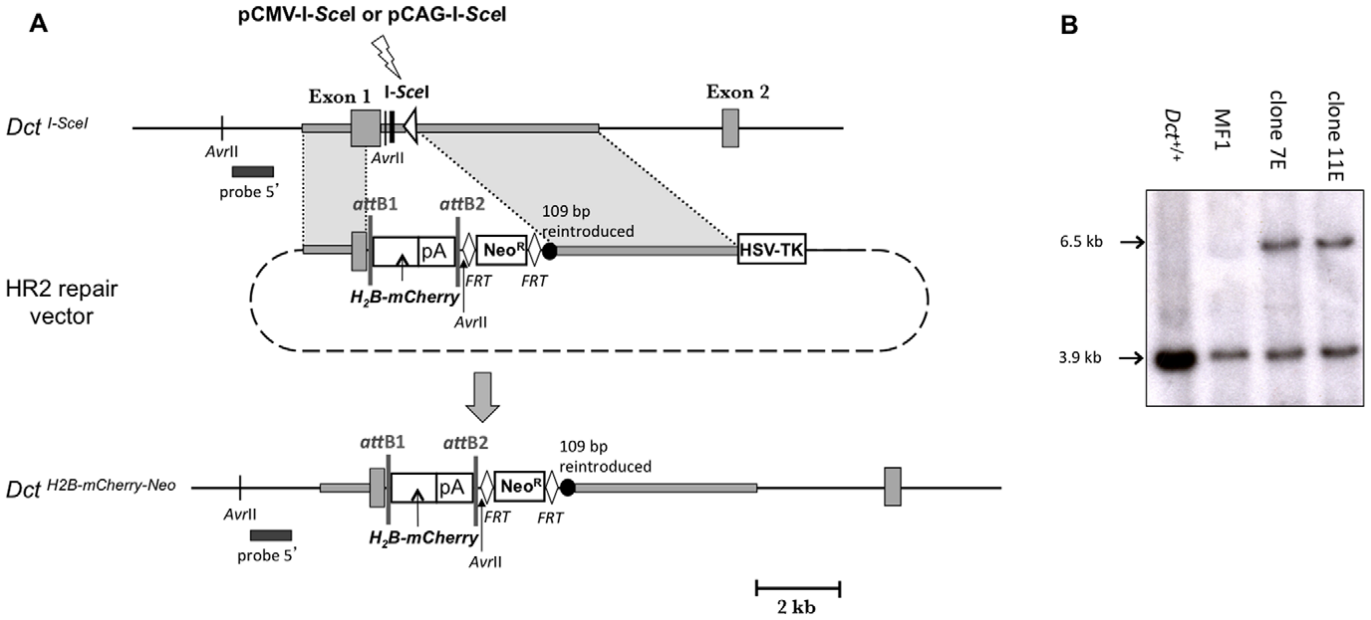
H_2_B-*mCherry* gene targeting by HR at the *Dct* locus. (A) Insertion of H_2_B-*mCherry* gene at the *Dct* locus. The *Dct^I-SceI^* allele, the HR2 repair vector and the *Dct^H2B^* ^-*mCherry*-*Neo*^ allele are represented from top to bottom. *Avr*II sites are indicated. There are no homologous sequences between *Dct^I-SceI^* and HR2 close to the I-*Sce*I site. A lightning denotes I-*Sce*I expression from pCMV-I-*Sce*I or pCAG-I-*Sce*I plasmid. The 1.4 and 4.5 kb of *Dct* isogenic DNA are depicted by grey rectangles. (B) Southern blot analysis of *Dct^+/+^*, *Dct ^I-SceI/+^*ES cells, and *Dct^H2B-mCherry-Neo/+^* ES clones 7E and 11E. ES clones 7E and 11E were obtained after transfection with the linear HR2 repair vector, and with both the supercoiled HR2 repair vector and pCAG-I-SceI expression plasmid, respectively. Genomic DNAs were digested with *Avr*II. The probe used for the hybridization is the external 5′ probe depicted by a black bar. The 3.9 kb fragment is carried by the *Dct ^+^* and *Dct ^I-SceI^* alleles, and the 6.5 kb fragment is distinctive of the *Dct ^H2B-mCherry-Neo^* targeted allele.

We thus decided to remove the two short regions of homology (including the *loxP* site) and to construct a novel repair vector. Therefore, a second destination vector (DV2) was produced using the H_2_B-*mCherry* reporter gene [31]. The HR2 repair vector was constructed (Fig. 2). It carries H_2_B-*mCherry*, a Neo^R^ cassette flanked with *FRT* sites, two regions of homology with the *Dct^I-SceI^* allele, 1.4 and 4.5 kb in length, and a HSV-TK negative selection cassette (Fig. 5A). By contrast with HR1, HR2 displays neither a short region of homology with the *Dct* gene next to the I-*Sce*I site nor a *loxP* site.

We assessed the rate of targeted insertion of H_2_B-*mCherry* at the *Dct* locus by I-*Sce*I-induced HR. MF1 ES cells were electroporated with supercoiled HR2 plasmid alone or with either pCMV-I-*Sce*I or pCAG-I-*Sce*I expression plasmids (Fig. 5A). As an additional control, MF1 ES cells were electroporated with linearized HR2 plasmid. The cells were cultured in the presence of G418 and gancyclovir. Colony counting revealed 397 and 370 colonies in presence of pCMV-I-*Sce*I and pCAG-I-*Sce*I plasmids respectively, and 653 colonies in the absence of the meganuclease (Table 1). Electroporation with linear HR2 plasmid, representative of a conventional gene targeting experiment, revealed 635 resistant colonies to both antibiotics. For each experiment, 144 colonies were individually picked up, amplified and PCR tested. Electroporation with linear HR2 plasmid resulted in one targeted clone (clone 7E). No targeted clones were seen in the supercoiled HR2-electroporated MF1 ES cells. The same results were obtained in the MF1 ES cells electroporated with both supercoiled HR2 and pCMV-I-*Sce*I plasmids. Electroporation with HR2 and pCAG-I-*Sce*I led to one targeted clone (clone 11E). Both conventional (linear HR2) and I-*Sce*I-mediated (supercoiled HR2 and pCAG-I-*Sce*I) gene targeting HR were confirmed by Southern blot analysis (Fig. 5B). Thus gene targeting using the HR2 repair vector and pCAG-I-*Sce*I led to a frequency of HR that could be estimated at 1.6×10^−7^ events per treated cell whereas conventional gene targeting led to a frequency of 2.7×10^−7^ events per treated cell. Therefore, the I-*Sce*I-induced DSB strategy does not seem to improve the frequency of HR at the *Dct* locus.

### Transient Expression of I-*Sce*I Triggers DSB in *Dct^I-Scei^* ^/+^ ES Cells

To test whether the meganuclease could indeed trigger DSB *in vivo* at the *Dct* locus in *Dct^I-SceI^* ^/+^ ES cells, we electroporated I-*Sce*I-expressing plasmids into MF1 ES cells and assayed the DNA lesion at the I-*Sce*I site using a sensitive technique, known as ligation-mediated PCR (LM-PCR), that allows the specific detection of breaks in a defined region of genomic DNA [34]. pCMV-I-*Sce*I, pCAG-I-*Sce*I and a mock plasmid were independently electroporated into MF1 ES cells and four hours later the genomic DNAs were extracted.

**Figure 6:**
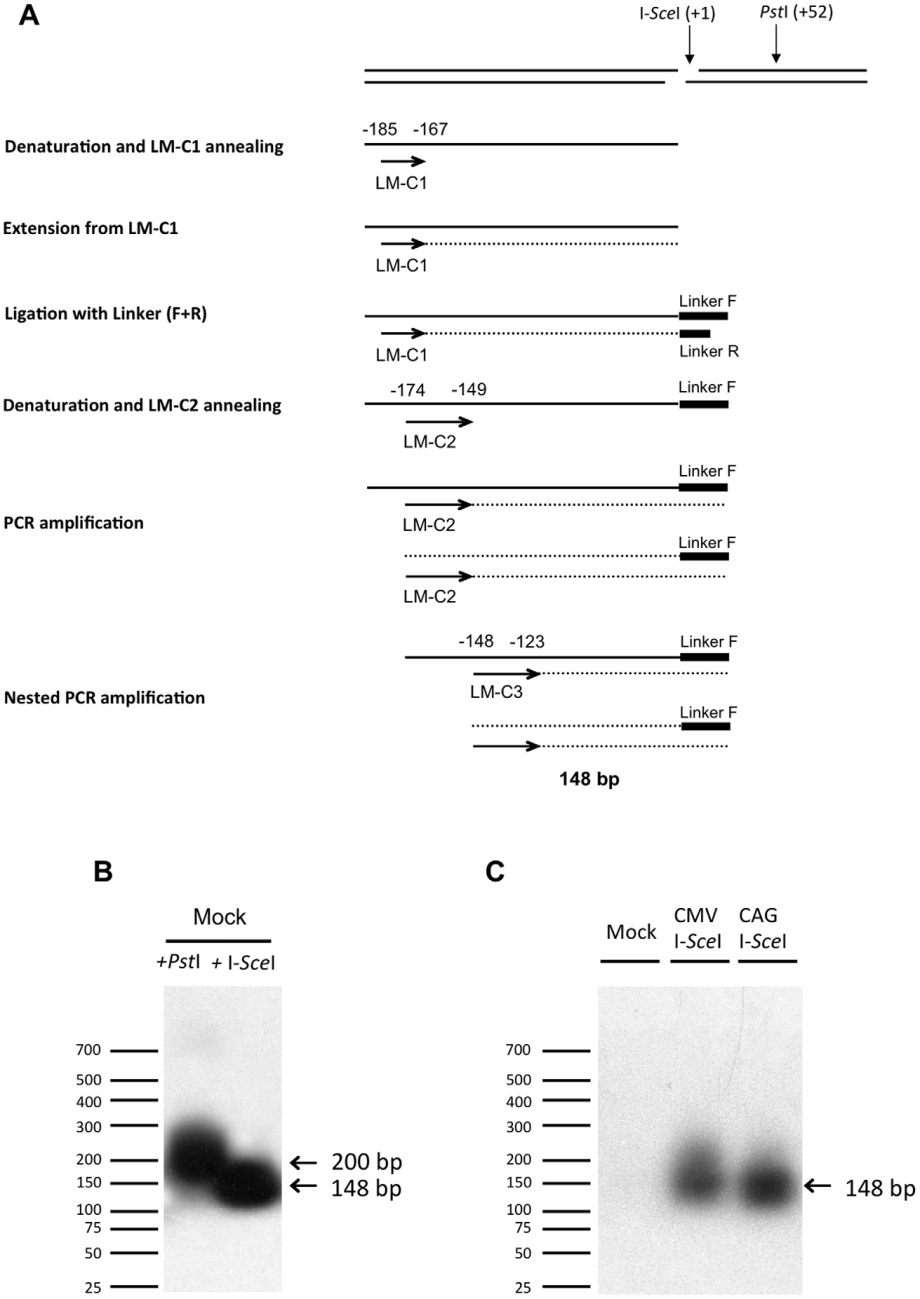
Assay of double-strand break induced by I-*Sce*I at the *Dct* locus. (A) Diagram of the ligation-mediated PCR (LM-PCR) technique to analyze a lesion at the I-*Sce*I site. The successive steps of LM-PCR are represented from top to bottom. Arrows indicate the *Dct* gene-specific primers LM-C1, LM-C2 and LM-C3. Two black bars depict the asymmetrical synthetic double-stranded linker constituted of linkerF and linkerR primers. After cleavage and denaturation of genomic DNA, LM-C1 primer was annealed and extended. Then, the double-stranded linker was ligated to the blunt-ended fragment. Fragments were PCR amplified using LM-C2 and linkerF primers. A second nested PCR amplification was performed using LM-C3 and linkerF primers. A lesion at the I-*Sce*I site would lead to a 148 bp LM-PCR product. (B) Detection of cleavage by the LM-PCR assay. Genomic DNA from mock-transfected MF1 ES cells was extracted, treated *in vitro* with *Pst*I (lane 1) or I-*Sce*I (lane 2), and analyzed by LM-PCR. After an autoradiographic exposure time of 2 h, both long and short PCR products (200 bp and 148 bp) are seen, as expected for *Pst*I and I-*Sce*I-treated DNA. The positions of size standards (in bp) are shown on the left. (C) Induction of DSBs by I-*Sce*I in ES cells. Genomic DNA from mock-transfected, pCMV-I-*Sce*I and pCAG-I-*Sce*I expression plasmid-transfected MF1 ES cells was extracted and analyzed by LM-PCR. After an autoradiographic exposure time of 16 h, no LM-PCR products is observed when DNA from mock-transfected cells was used as a template. A fragment of the predicted 148 bp size is seen after LM-PCR-amplification of DNA from cells transfected with pCMV-I-*Sce*I and pCAG-I-*Sce*I. The positions of size standards (in bp) are shown on the left.

In a first step, we tested the specificity and sensibility of the LM-PCR on transfected ES cells. We used two sites recognized by restriction endonucleases: (i) the I-*Sce*I site, whose cleavage was under evaluation; (ii) a *Pst*I site at position +52 relative to the I-*Sce*I site. Approximately 2 µg of extracted genomic DNA from mock plasmid-transfected *Dct^I-SceI/+^* MF1 ES cells were digested with *Pst*I or I-*Sce*I restriction endonuclease respectively. Then the digested DNA was heated to allow annealing with a first *Dct* gene-specific primer (LM-C1) located at position -185 relative to the I-*Sce*I site. This was followed by LM-C1 primer extension that terminated at the site of a break to produce a blunt-ended DNA, which was then ligated to an asymmetrical synthetic double-stranded linker. The newly synthesized DNA molecule was denatured to allow annealing with a second *Dct* gene-specific primer (LM-C2) located at position -174 relative to the I-*Sce*I site and amplification in a PCR reaction with linker primer. The PCR-amplified products were exponentially amplified by nested PCR using a third *Dct*-gene-specific primer (LM-C3) located at position −148 relative to the I-*Sce*I site and linker primer, as shown in Fig. 6A. Finally, the PCR products were separated on an agarose gel, alkaline blotted to a nylon membrane, and hybridized with a radioactive probe which does not overlap the primer sequences. PCR products of the predicted sizes, 148 bp for I-*Sce*I digestion and 200 bp for *Pst*I digestion, were seen (Fig. 6B). These data indicate that the LM-PCR technique allowed the specific detection of a cleavage generated *in vitro* on the genomic DNA from MF1 ES cells.

In a second step, we evaluated the ability of the meganuclease to trigger a DSB *in vivo* at the *Dct* locus. Approximately 2 µg of extracted genomic DNA from mock plasmid-transfected MF1 ES cells, pCMV-I-*Sce*I- transfected MF1 ES cells and pCAG-I-*Sce*I-transfected MF1 ES cells were directly analyzed by LM-PCR. Figure 6C shows that no DNA lesions occurred at significant level at the I-*Sce*I site in the absence of I-*Sce*I expression. When the genomic DNA from pCMV-I-*Sce*I-transfected MF1 ES cells was used as a template in the LM-PCR reaction, a 148 bp amplification product was detected, showing that expression of the meganuclease in MF1 ES cells triggered DSB at the I-*Sce*I restriction site. LM-PCR in which the genomic DNA from pCAG-I-*Sce*I-transfected cells was used as a template produced similar results (Fig. 6C), suggesting that both I-*Sce*I expression vectors were equally efficient in triggering DSB at the target locus.

## Discussion

In this report, we provided evidence that I-*Sce*I-induced DSB in ES cells does not improve the efficacy of the gene targeting methodology at the *Dct* locus compared to the conventional approach. Electroporation was used to introduce the I-*Sce*I expressing plasmids into ES cells and a low efficiency of transfection could explain these results. However, we were able to detect the expression of enhanced green fluorescent protein (GFP) from *Aequora victoria* by fluorescence-activated cell sorter (FACS) analysis in 65% of a population of CK35 ES cells electroporated with a plasmid containing the CMV promoter driving the expression of the GFP, indicating efficient electroporation (data not shown). These data agree with a previous report [35]. Both repair vectors (HR1 and HR2) contain 5.9 kb of *Dct* homology. The same homology was used previously by Guyonneau et al. [23] to inactivate the *Dct* gene in ES cells, indicating that such a length is efficient for a gene replacement event. However, we cannot exclude that increasing the length of homology may improve I-*Sce*I-mediated HR at the *Dct* locus. Since the first repair vector (HR1) contained short regions of homology in the vicinity of the I-*Sce*I site, we hypothesized that these regions were preferentially used to repair the DSB, thus generating homologous recombinant clones that did not integrate the Neo^R^ cassette and died eventually in the presence of G418. Therefore, a second repair vector (HR2) with no homology to the sequence surrounding the I-*Sce*I site was generated, but we still failed to demonstrate improvement of the frequency of gene targeting.

Previous experiments suggested that non-homologous recombination may be more efficient than plasmid-to-chromosome HR to repair a chromosomal DSB introduced by I-*Sce*I. Indeed, when mouse Ltk^-^ fibroblasts carrying a selectable herpes simplex virus thymidine kinase (*tk*) gene mutated by the 18-bp I-*Sce*I site, were electroporated with I-*Sce*I meganuclease and a repair plasmid with the functional *tk* gene, *tk^+^* clones were recovered. However, all analyzed *tk^+^* cells contained deletions that restored the reading frame of the *tk* gene, indicating that the recovery of a functional *tk* gene did not occur through HR of the integrated *tk* gene with a transfected *tk* fragment, but rather via resection and ligation [36]. These data were obtained in mouse Ltk^−^ fibroblasts, not in ES cells. This deserves mention since distinct differences in frequencies of targeted integration driven by a DSB among cell types have been reported. HR after cleavage by a zinc-finger nuclease (ZFN) at the *CCR5* locus in presence of cognate donor linear and circular episomes was more efficient in a panel of immortalized cell lines from human leukemia than in human stem cells, such as cord blood CD34^+^ hematopoietic cells and human ES cells [37]. It has also been reported that the rate of ZFN-mediated gene targeting at the *Rosa26* locus was higher in primary fibroblasts from adult mice than in murine ES cells [38]. These results suggest that DSB-induced gene targeting may be lower in ES cells than in somatic cells. This contention seems inconsistent with reports showing that HR is the predominant pathway to repair DSBs in ES cells, whereas somatic cells utilize non-homologous end joining (NHEJ) [39], [40]. It has also been reported that ES cells that had been allowed to differentiate preferred the error-prone NHEJ pathway to the high-fidelity HR to repair DNA DSBs [39]. Because ES cells and somatic cells are intrinsically different in the extent to which they preserve their genomic integrity [41], it was important to assess that our experiments were made with genuine ES culture rather than differentiated ES culture. We confirmed that the *Dct^I−SceI/+^* CK35 cells are truly pluripotent ES cells, able to colonize the germ line. Furthermore, CK35 ES cells have been previously used to demonstrate highly efficient gene targeting after DSB [3].

We observed that the repair vector was not inserted at the *Dct* locus in the majority of neomycin- and gancyclovir-resistant clones. One possible explanation was that I-*Sce*I did not cleave its recognition sequence in *Dct^I−SceI/+^* ES cells. However, we showed here the existence of I-*Sce*I-induced DSBs at the *Dct* locus using LM-PCR. This result does not rule out the possibility that the efficiency of cleavage by I-*Sce*I is an important factor to favor HR at a given individual locus. Recent findings by Daboussi et al. [42] support this conclusion. They found that chromatin accessibility modulates the ability of meganucleases to induce targeted gene modification in human 293-H cells.

Seminal experiments of gene targeting in ES cells based on an I-*Sce*I-induced gene replacement system were first performed with mutated resistance genes integrated in chromosomal sequences [8], and later extended to natural endogenous genes, *Hprt*, *Villin* and *Dbx1* genes [3], [4], [43]. Importantly, random integrations could not be detected in the previous studies performed with I-*Sce*I in mouse ES cells when selection strategies were specifically designed to single out the gene targeting events and eliminate the nonrecombinant recombination events [3], [4], [8]. Altogether, the number of actual genes that have been efficiently targeted following I-*Sce*I-mediated HR in ES cells is still limited. It is widely accepted that the efficiency of conventional HR depends on the target locus. Our results suggest that, similarly, efficiency of gene correction by DSB-induced HR may be highly dependent on the targeted locus. We anticipate that deeper analysis of the meganuclease, repair vector, target locus and cells that do not show enhanced HR by DSB may also shed light on the nature of the factors that contribute to gene targeting in mammalian cells.

## References

[1] Vasquez KM, Marburger K, Intody Z, Wilson JH (2001). Manipulating the mammalian genome by homologous recombination.. Proceedings of the National Academy of Sciences of the United States of America.

[2] Choulika A, Perrin A, Dujon B, Nicolas JF (1995). Induction of homologous recombination in mammalian chromosomes by using the I-SceI system of Saccharomyces cerevisiae.. Molecular and cellular biology.

[3] Cohen-Tannoudji M, Robine S, Choulika A, Pinto D, El Marjou F (1998). I-SceI-induced gene replacement at a natural locus in embryonic stem cells.. Molecular and cellular biology.

[4] Donoho G, Jasin M, Berg P (1998). Analysis of gene targeting and intrachromosomal homologous recombination stimulated by genomic double-strand breaks in mouse embryonic stem cells.. Molecular and cellular biology.

[5] Liang F, Han M, Romanienko PJ, Jasin M (1998). Homology-directed repair is a major double-strand break repair pathway in mammalian cells.. Proceedings of the National Academy of Sciences of the United States of America.

[6] Rouet P, Smih F, Jasin M (1994). Introduction of double-strand breaks into the genome of mouse cells by expression of a rare-cutting endonuclease.. Molecular and cellular biology.

[7] Sargent RG, Brenneman MA, Wilson JH (1997). Repair of site-specific double-strand breaks in a mammalian chromosome by homologous and illegitimate recombination.. Molecular and cellular biology.

[8] Smih F, Rouet P, Romanienko PJ, Jasin M (1995). Double-strand breaks at the target locus stimulate gene targeting in embryonic stem cells.. Nucleic acids research.

[9] Izmiryan A, Basmaciogullari S, Henry A, Paques F, Danos O (2011). Efficient gene targeting mediated by a lentiviral vector-associated meganuclease.. Nucleic acids research.

[10] Gong WJ, Golic KG (2003). Ends-out, or replacement, gene targeting in Drosophila.. Proceedings of the National Academy of Sciences of the United States of America.

[11] Puchta H, Dujon B, Hohn B (1996). Two different but related mechanisms are used in plants for the repair of genomic double-strand breaks by homologous recombination.. Proceedings of the National Academy of Sciences of the United States of America.

[12] Steel KP, Davidson DR, Jackson IJ (1992). TRP-2/DT, a new early melanoblast marker, shows that steel growth factor (c-kit ligand) is a survival factor.. Development.

[13] Pavan WJ, Tilghman SM (1994). Piebald lethal (sl) acts early to disrupt the development of neural crest-derived melanocytes.. Proceedings of the National Academy of Sciences of the United States of America.

[14] Nishimura EK, Jordan SA, Oshima H, Yoshida H, Osawa M (2002). Dominant role of the niche in melanocyte stem-cell fate determination.. Nature.

[15] Mackenzie MA, Jordan SA, Budd PS, Jackson IJ (1997). Activation of the receptor tyrosine kinase Kit is required for the proliferation of melanoblasts in the mouse embryo.. Developmental biology.

[16] Aubin-Houzelstein G, Djian-Zaouche J, Bernex F, Gadin S, Delmas V (2008). Melanoblasts' proper location and timed differentiation depend on Notch/RBP-J signaling in postnatal hair follicles.. The Journal of investigative dermatology.

[17] Guyonneau L, Rossier A, Richard C, Hummler E, Beermann F (2002). Expression of Cre recombinase in pigment cells.. Pigment cell research.

[18] Dunn KJ, Brady M, Ochsenbauer-Jambor C, Snyder S, Incao A (2005). WNT1 and WNT3a promote expansion of melanocytes through distinct modes of action.. Pigment cell research.

[19] Lanning JL, Wallace JS, Zhang D, Diwakar G, Jiao Z (2005). Altered melanocyte differentiation and retinal pigmented epithelium transdifferentiation induced by Mash1 expression in pigment cell precursors.. The Journal of investigative dermatology.

[20] Woods SL, Bishop JM (2011). A new transgenic mouse line for tetracycline inducible transgene expression in mature melanocytes and the melanocyte stem cells using the Dopachrome tautomerase promoter.. Transgenic research.

[21] Pollock PM, Cohen-Solal K, Sood R, Namkoong J, Martino JJ (2003). Melanoma mouse model implicates metabotropic glutamate signaling in melanocytic neoplasia.. Nat Genet.

[22] Zhao S, Overbeek PA (1999). Tyrosinase-related protein 2 promoter targets transgene expression to ocular and neural crest-derived tissues.. Developmental biology.

[23] Guyonneau L, Murisier F, Rossier A, Moulin A, Beermann F (2004). Melanocytes and pigmentation are affected in dopachrome tautomerase knockout mice.. Molecular and cellular biology.

[24] Egidy G, Jule S, Bosse P, Bernex F, Geffrotin C (2008). Transcription analysis in the MeLiM swine model identifies RACK1 as a potential marker of malignancy for human melanocytic proliferation.. Mol Cancer.

[25] Winnepenninckx V, Lazar V, Michiels S, Dessen P, Stas M (2006). Gene expression profiling of primary cutaneous melanoma and clinical outcome.. J Natl Cancer Inst.

[26] Choulika A, Perrin A, Dujon B, Nicolas JF (1994). The yeast I-Sce I meganuclease induces site-directed chromosomal recombination in mammalian cells.. Comptes rendus de l'Academie des sciences Serie III, Sciences de la vie.

[27] Okabe M, Ikawa M, Kominami K, Nakanishi T, Nishimune Y (1997). 'Green mice' as a source of ubiquitous green cells.. FEBS letters.

[28] Kress C, Vandormael-Pournin S, Baldacci P, Cohen-Tannoudji M, Babinet C (1998). Nonpermissiveness for mouse embryonic stem (ES) cell derivation circumvented by a single backcross to 129/Sv strain: establishment of ES cell lines bearing the Omd conditional lethal mutation.. Mammalian genome.

[29] Robertson E, Bradley A, Kuehn M, Evans M (1986). Germ-line transmission of genes introduced into cultured pluripotential cells by retroviral vector.. Nature.

[30] Budd PS, Jackson IJ (1995). Structure of the mouse tyrosinase-related protein-2/dopachrome tautomerase (Tyrp2/Dct) gene and sequence of two novel slaty alleles.. Genomics.

[31] Abe T, Kiyonari H, Shioi G, Inoue K, Nakao K (2011). Establishment of conditional reporter mouse lines at ROSA26 locus for live cell imaging.. Genesis.

[32] Gu H, Zou YR, Rajewsky K (1993). Independent control of immunoglobulin switch recombination at individual switch regions evidenced through Cre-loxP-mediated gene targeting.. Cell.

[33] Elliott B, Richardson C, Winderbaum J, Nickoloff JA, Jasin M (1998). Gene conversion tracts from double-strand break repair in mammalian cells.. Molecular and cellular biology.

[34] Besaratinia A, Pfeifer GP (2009). DNA-lesion mapping in mammalian cells.. Methods.

[35] Ward CM, Stern PL (2002). The human cytomegalovirus immediate-early promoter is transcriptionally active in undifferentiated mouse embryonic stem cells.. Stem Cells.

[36] Lukacsovich T, Yang D, Waldman AS (1994). Repair of a specific double-strand break generated within a mammalian chromosome by yeast endonuclease I-SceI.. Nucleic acids research.

[37] Lombardo A, Genovese P, Beausejour CM, Colleoni S, Lee YL (2007). Gene editing in human stem cells using zinc finger nucleases and integrase-defective lentiviral vector delivery.. Nature biotechnology.

[38] Connelly JP, Barker JC, Pruett-Miller S, Porteus MH (2010). Gene correction by homologous recombination with zinc finger nucleases in primary cells from a mouse model of a generic recessive genetic disease.. Molecular therapy.

[39] Tichy ED, Pillai R, Deng L, Liang L, Tischfield J (2010). Mouse embryonic stem cells, but not somatic cells, predominantly use homologous recombination to repair double-strand DNA breaks.. Stem cells and development.

[40] Serrano L, Liang L, Chang Y, Deng L, Maulion C (2011). Homologous recombination conserves DNA sequence integrity throughout the cell cycle in embryonic stem cells.. Stem cells and development.

[41] Stambrook PJ, Tichy ED (2010). Preservation of genomic integrity in mouse embryonic stem cells.. Advances in experimental medicine and biology.

[42] Daboussi F, Zaslavskiy M, Poirot L, Loperfido M, Gouble A (2012). Chromosomal context and epigenetic mechanisms control the efficacy of genome editing by rare-cutting designer endonucleases. Nucleic acids research.. In press.

[43] Bielle F, Griveau A, Narboux-Neme N, Vigneau S, Sigrist M (2005). Multiple origins of Cajal-Retzius cells at the borders of the developing pallium.. Nature neuroscience.

